# Coarse Grained Molecular Dynamics Simulations of Transmembrane Protein-Lipid Systems

**DOI:** 10.3390/ijms11062393

**Published:** 2010-06-09

**Authors:** Peter Spijker, Bram van Hoof, Michel Debertrand, Albert J. Markvoort, Nagarajan Vaidehi, Peter A.J. Hilbers

**Affiliations:** 1 Department of Biomedical Engineering, Eindhoven University of Technology, P.O. Box 513, 5600 MB, Eindhoven, The Netherlands; 2 Division of Immunology, Beckman Research Institute of the City of Hope, 1500 E. Duarte Road, Duarte, CA-91010, USA

**Keywords:** molecular dynamics, coarse grained, transmembrane proteins, α-helices, WALP-peptides, hydrophobic mismatch

## Abstract

Many biological cellular processes occur at the micro- or millisecond time scale. With traditional all-atom molecular modeling techniques it is difficult to investigate the dynamics of long time scales or large systems, such as protein aggregation or activation. Coarse graining (CG) can be used to reduce the number of degrees of freedom in such a system, and reduce the computational complexity. In this paper the first version of a coarse grained model for transmembrane proteins is presented. This model differs from other coarse grained protein models due to the introduction of a novel angle potential as well as a hydrogen bonding potential. These new potentials are used to stabilize the backbone. The model has been validated by investigating the adaptation of the hydrophobic mismatch induced by the insertion of WALP-peptides into a lipid membrane, showing that the first step in the adaptation is an increase in the membrane thickness, followed by a tilting of the peptide.

## 1. Introduction

The cell membrane maintains the essential differences between the cytoplasmic and the extracellular environment and additionally has the task to transport signals or substances from one side to the other. By doing so, the membrane is a rather complex system on its own, consisting mainly of lipid molecules and membrane proteins. How a membrane protein is associated with the lipid membrane reflects the function of the protein. Transmembrane proteins can function on both sides of the membrane or transport molecules across it. Cell-surface receptors are transmembrane proteins that bind water-soluble signal molecules in the extracellular space and generate different intracellular signals.

The largest family of cell surface receptors is formed by the G-protein coupled receptors (GPCRs), which are responsible for most transmembrane signal transduction by hormones and neurotransmitters, as well as for the senses of vision, smell and taste [1–3]. All of the GPCRs belong to a large family of homologous, seven-pass transmembrane proteins. The interaction between the receptor and its target protein is mediated by a third protein, a trimeric GTP-binding protein (G-protein). When a ligand binds to the extracellular domain of the GPCR, the receptor undergoes a conformational change that enables the activation of the G-protein [4].

The historical lack of 3D crystal structures has lead to the development of different computational methods in order to predict the structure of a GCPR [5–7]. More recently, the activation pathway of different GPCR complexes have been investigated using molecular dynamics (MD), where the solvent and membrane environment is taken into account [8–11].

Using MD simulation techniques at the atomistic level allows for the exploration of ligand positioning and the initialization of protein activation. However, due to the computational demand, it is not feasible to model the entire cascade of protein activation with atomistic detail. To partially overcome these limitations it is possible to use a coarse grained (CG) model instead of the atomistic one.

With a CG model typically several atoms are grouped into one particle, while the interaction properties of the new particle are modeled as close as possible to those of the original atoms it represents. By grouping atoms together, coarse graining reduces the degrees of freedom and, therefore, the computational cost. Moreover, due to the removal of high-frequency motions, such as the vibrational movements of hydrogen atoms, larger steps can be made in MD simulations, allowing longer simulations. Simultaneously, the dynamics in the CG system is likely to be further increased as additional friction and noise from eliminated degrees of freedom are absent. Still, an important point with coarse graining a system is with determining the effective interactions between the CG particles.

One of the first applications of CG methods has been the MD simulation of the aggregation of amphiphiles [12]. Since then many different applications for coarse graining have been studied, ranging from polymer chains [13–19] to lipid systems [20–24]. We have used lipid CG methods to study many interesting properties of bilayer systems, such as the spontaneous formation of vesicles [23], the fusion and fission of vesicles [25–27], as well as shape changes of vesicles due to changing conditions in the environment [28,29].

More recently coarse graining methods have been applied to more complex systems such as small peptides or even larger proteins [30–33], as well as viral capsids [34,35]. However, all coarse graining methods differ in their approach with respect to the number of atoms per CG particle as well as in the way interactions are described. In this paper the first version of a new CG model for proteins is being developed, which differs from other models due to the introduction of a novel angle potential with two equilibrium angles as well as a hydrogen bonding potential. These new potentials are used to stabilize the backbone without the need to use different particle types for different parts of the protein backbone. In this approach we differ from other CG models of transmembrane proteins in which one on forehand has to decide for each backbone particle type which are, for instance, in a helical or in a loop conformation [32,33,36,37]. It must be emphasized that the model presented in this paper is still in development and inclusion of new features and reparametrization are likely to occur. Moreover, the main focus of the model is on a qualitative description of transmembrane peptides and proteins (consisting mainly of *α*-helices), but the CG model is also tested against globular proteins. Finally, to prove the possible applicability of the new CG model, it is used to investigate membrane inserting properties of a large set of WALP-peptides. All MD simulations in this paper are performed using our in-house developed code [23].

## 2. Computational Model

As mentioned in the introduction, one of the steps involved in designing a CG model is to choose the mapping of the original atoms to the CG particles. Different levels of coarse graining are possible, ranging from only omitting the hydrogen atoms (the united atoms approach) to much higher level CG particles combining several original atoms. However, the most common approach is to group four non-hydrogen atoms into one CG particle [20,22,23]. Thus, for instance in the case of lipid membranes, dipalmitoylphosphatidylcholine (DPPC) is represented by four particles in the head group (representing the choline, phosphate and glycerol backbone) and two hydrocarbon tails, each consisting of four tail particles.

**Figure 1:**
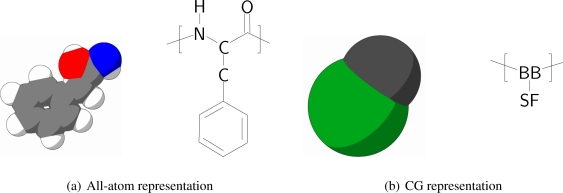
Mapping of the all-atom representation of phenylalanine (figures to the left, both schematic and as chemical composition) to its CG representation (figures to the right). The center of the CG backbone particle is determined by the position of the C*_α_* atom, whereas the CG sidechain particle is positioned along the C*_α_*-C*_β_* bond vector. The brackets represent the repetitive unit.

In order to keep the CG protein particles in the new protein model in the same range of mass and size with respect to the CG bilayer models, amino acids are mapped into two CG interaction sites, one for the backbone and one for the sidechain. The backbone particle is located at the position of C*_α_* in the all-atom model, whereas the sidechain particle is located along the C*_α_*-C*_β_* bond vector, with its specific position depending on the specific residue type of the amino acid, which is discussed further on in the paper. The size of the CG particles resembles the size of the underlying original atoms. In Figure 1 the mapping of phenylalanine to the CG model is schematically depicted.

**Figure 2:**
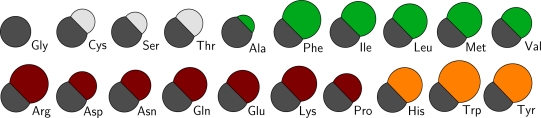
A schematic overview of all the residue types used in the CG protein model. The sizes of all particles are proportional with respect to each other. All residues (except for Gly) can be assigned to any of the four groups (as discussed in the text): the neutral group (Cys, Ser, Thr), the apolar group (Ala, Phe, Ile, Leu, Met, Val), the polar group (Arg, Asp, Asn, Gln, Glu, Lys, Pro), and the aromatic group (His, Trp, Tyr). Each of these groups is indicated by a different color.

The mass of all particles is computed directly from the chemical composition and the size of the particles is computed from the occupied volumes of the residues [38]. Because of the chosen spherical nature of the CG particles, the Van derWaals radius of each particle is computed directly from its volume. The radii of the backbone particles are all assumed to be equal to that calculated from glycine. To obtain the volumes of the sidechain particles of the other residues, the volume of glycine is subtracted from the total residue volume. Since no occupied volume for arginine has been reported, its volume is estimated to be equal to that of phenylalanine which has an equal number of non-hydrogen atoms. This method to obtain the size of the CG particles is similar to that used by Levitt [39]. However, different to Levitt, it is chosen to use particles that are less soft, with respect to their interactions, in order to make them more compatible with existing water and lipid CG models. In Figure 2 a schematic overview of the sizes of all residue types in the CG protein model is depicted, and in Table S1 of the supplementary material the masses and sizes of all CG particles are given, including the lipid and water parameters from the lipid model by Markvoort *et al.* [23].

All CG protein particle types are denoted either by their full or abbreviated name (e.g., isoleucine or Ile) or by two letters, where the backbone particle notation starts with a B, and the sidechain particle notation with a S. Depending on the type of amino acid the sidechain particle resembles, the second letter is added in accordance with the one letter abbreviation for the residue (thus, SF for phenylalanine). For the backbone particle three types are distinguished, only differing in mass: the default type (BB) and two types to account for the proline residue (BP) and the glycine residue (BG, which has no sidechain particle attached).

### 2.1. Force Field Development

Besides the topological mapping of the original atoms into CG particles, it is also necessary to derive interaction parameters (the force field) for all of the CG particles; both bonded and non-bonded interactions. This is a difficult and tedious process, since the problem is highly multi-dimensional.

The general force field equation that describes all contributions to the potential energy *U* of the coarse grained system is given by

(1) $$V_{\text{total}}=\sum_{}^{N_{\text{pairs}}}V_{\text{LJ}}(r_{ij})+\sum_{}^{{N^{\prime }}_{\text{pairs}}}V_{\text{LJHB}}(r_{ij})+\sum_{}^{N_{\text{bonds}}}V_{\text{harm}}(r_{ij})+\sum_{}^{N_{\text{angles}}}V_{\text{harm}}(\theta_{ijk})+\sum_{}^{N_{\text{double}\;\text{angles}}}V_{\text{double}}(\theta_{ijk})$$

where the first two sums over all pairs (or a subset, indicated by the prime, as explained further on in this paper) in the system account for the nonbonded interactions (a standard Lennard-Jones (LJ) potential or modified version (LJHB) as explained further on), the third sum all interactions due to atoms that are bonded (a standard harmonic potential), and the fourth and fifth sums for all possible bonded angle triplets (either a standard harmonic or double angle potential). Which angles are subject to which potentials (harmonic or double angle) is explained further on in this paper.

In the current CG model for the bonded interactions only bond and angle terms are considered, both in their harmonic form. Torsion interactions, either dihedral or improper, are not present in the model, in contrast to other CG protein models [33,40]. Although torsion interactions are normally used in order to stabilize the secondary structure of the protein, using these interactions also means that it has to be decided on forehand by using particle typing which part of the protein is going to be, for instance, helical, extended or in a loop. Therefore, the torsional interactions are discarded in the current model, but in order to account for the ability to maintain the secondary structure a force field extension is developed for non-bonded interactions, which is explained further on.

First the parameters of the CG force field with respect to the bonded interactions are discussed, followed by a discussion for the non-bonded interactions. Parameters for lipid and water molecules are taken from an existing bilayer model [23]. Because the new CG transmembrane protein model is based upon Markvoort’s bilayer model, the time scale of diffusion of molecules in the system is of similar size, leading to a similar time scale for the simulations [25].

### 2.2. Bonded Interactions

Average bond lengths for CG backbone particles are determined from all-atom MD simulations of several nanoseconds of both rhodopsin [41] and the *β*_2_AR [8]. For both trajectories the all-atom representation of the backbone has been mapped into the CG representation. Subsequently, the distances between neighboring CG backbone particles are determined. This gives a probability distribution for the bond length for CG backbone particles with an average of 0.384 nm. Since both simulations are of transmembrane proteins (consisting mainly of *α*-helices), the bond length distribution could be biased toward *α*-helices. Because the main goal is to develop a CG transmembrane protein model the bias is acceptable. Furthermore, the computed bond length is in good agreement with previous studies where the same CG bond length is based on crystal structures of many different proteins found in the protein data bank [39,42].

Unfortunately, the all-atom simulations do not hold sufficient data to accurately determine the bond lengths between CG backbone particles and sidechain particles. Therefore, these bond lengths are based on previously determined bond lengths by averaging across entries in the protein data bank [43]. The bond length associated with the disulfide bond has been obtained from the previously mentioned all-atom MD simulations.

Bond strengths are initially based on the constants reported by Wallqvist [42] and, for the disulfide bond, by Levitt [39]. However, because in the current model different masses are used, the constants have been adjusted in such a way, that the bond oscillation frequencies are the same. The bond lengths and force constants are listed in Table S2, which can be found in the supplementary materials.

In order to incorporate rigidity and allow for the anti conformation of the hydrocarbon tail the CG lipid model only uses harmonic angle interactions between tail particles. Thus, the equilibrium angle for lipid particles is set to 180°, with a force constant of 5.408 kJ/mol/rad^2^. The harmonic angle potential allows only one equilibrium angle to be set. Thus, the angle along the backbone of the protein would also only have one equilibrium angle using this potential. However, the backbone of a protein can be in very different conformations, for instance as *α*-helices or random coils, for which the angles along the backbone are very different (roughly around 90° and 120° respectively). Introducing different particle types, with different angle potentials between them, for each of these two possible conformations, would allow the different conformations, but it would also impose a-priori knowledge upon the model. A different approach is to allow two equilibrium angles in the potential energy function for the angle, and thus allow different conformations using only one particle type. To this end a novel angle potential with two equilibrium angles has been developed and is named the double angle potential.

**Figure 3:**
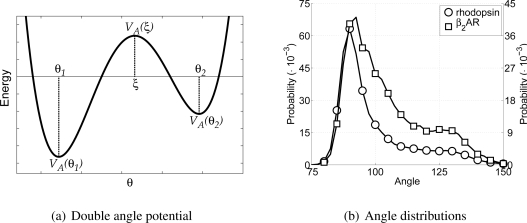
In (a) a schematic depiction of the double angle potential is shown and in (b) the distribution of angles along the backbone chain as found in all-atom MD simulations for rhodopsin and the *β*_2_AR are depicted. The distribution for rhodopsin is on the left axis and for the *β*_2_AR on the right axis.

The double angle potential is defined as a fourth power polynomial, which is determined by five parameters given in the force field parameter set. These five parameters are: the first equilibrium angle *θ*_1_, the second equilibrium angle *θ*_2_, the potential energy for the first equilibrium angle *V*_A_ (*θ*_1_), the potential energy for the second equilibrium angle *V*_A_ (*θ*_2_), and the maximal potential energy for the barrier between the two equilibrium angles *V*_A_ (*ξ*), where *ξ* is the angle belonging to the maximum of the barrier. It must be noted that not *ξ*, but the height of the maximum is given in the force field parameter set. In Figure 3(a) the general shape of the double angle potential is shown, indicating all parameters discussed above. Others have also used fourth power polynomials to describe similar angles, but either with equal well depths [42] or with only one equilibrium angle [44].

The functional form of the double angle potential is given by

(2) $$V_{\text{A}}(\theta_{ijk})=A_{g}(\theta_{ijk})+D$$

where *A* and *D* are both constants given by

(3) $$A=\frac{V_{\text{A}}(\xi )-V_{\text{A}}(\theta_{1})}{g(\xi )-g(\theta_{1})}$$

and

(4) $$D=\frac{g(\theta_{1})V_{\text{A}}(\xi )-g(\xi )V_{\text{A}}(\theta_{1})}{g(\theta_{1})-g(\xi )}$$

and *g* (*θ_ijk_*) the fourth power polynomial expressed as

(5) $$g(\theta_{ijk})=\frac{1}{4}\theta_{ijk}^{4}-\frac{1}{3}(\theta_{1}+\theta_{2}+\xi )\theta_{ijk}^{3}+\frac{1}{2}(\theta_{1}\theta_{2}+\theta_{1}\xi +\theta_{2}\xi )\theta_{ijk}^{2}-(\theta_{1}\theta_{2}\xi )\theta_{ijk}$$

Furthermore, the angle belonging to the maximum of the barrier obeys the requirement *θ*_1_ < *ξ* < *θ*_2_ and the energies for the minima satisfy *V*_A_ (*ξ*) > *V*_A_ (*θ*_1_) and *V*_A_ (*ξ*) > *V*_A_ (*θ*_2_). The value for *ξ* can be determined from all known parameters using the iterative Newton–Raphson method, which requires only a few steps under normal conditions. In the supplementary materials a complete derivation of the double angle potential is given.

Similar to determining the bond parameters for the CG particles, both all-atom MD simulations are used again to extract the probability distribution for the angles between CG backbone particles, see Figure 3(b). Furthermore, the Boltzmann inversion method [14,16,45] has been used to obtain estimates for values of the potential energy at any of the three angles. For the CG backbone particles this gives the equilibrium angles at 91.25° and 123.25°, with potential energies of 0 kJ/mol and 23.0 kJ/mol, respectively. By default the lowest energy in the double angle potential is set to zero, because the offset of the potential is arbitrary. The barrier energy is set at 23.7 kJ/mol. From these choices it can be seen that the model is developed for *α*-helices, mainly because the equilibrium angle around 90° is highly favored.

The angle between CG sidechain particles and two backbone particles (a B-B-S angle) is also governed by the double angle potential. This angle is introduced to restrain the movement of the sidechain particles. The two equilibrium angles are set such that the backbone-sidechain bond is the bisector outward of the underlying angle in the backbone. Thus, the equilibrium angles are chose to be 118° and 135°, both with a corresponding zero potential value. The barrier in between is very small (only 0.003 kJ/mol) allowing sidechain particles to adapt their position along the backbone relatively easy. All angle parameters are listed in Table S3 in the supplementary materials.

### 2.3. Non-Bonded Interactions

To account for interactions between CG particles belonging to different molecules and for long range interactions within a molecule not dealt with by the bonded interactions, an accurate description of non-bonded interactions is necessary. Between all CG particles, except those that are directly bonded or connected through an angle, both Van der Waals and Coulomb interactions are modeled by the Lennard–Jones potential [23]. When only repulsive interactions are to be taken into account an adaptation of the Lennard-Jones potential is used: the Weeks–Chandler–Andersen potential [46], which is effectively a Lennard-Jones potential truncated at the point of minimal energy and shifted to remain continuous. For distances larger than the truncation distance the potential is zero.

Within the CG protein model three non-bonded interaction classes can be distinguished: protein-protein, environment-protein, and environment-environment. Since the model is developed for transmembrane proteins, the latter interaction class consists mainly of lipid and water interactions. For the interactions between lipid and water particles the force field given by Markvoort *et al.* [23] is used.

The second interaction class, environment-protein, deals with all interactions of the lipids and water with the CG protein particles. These interactions are especially important in the case of transmembrane proteins, since in that case the environment is heterogeneous. Ulmschneider *et al.* have derived implicit potentials for amino acids in a membrane [47]. In their work a potential of mean force for each amino acid is derived from statistical data on the occurrence of the amino acid in the membrane region of the transmembrane proteins. This splits all amino acids into four clearly distinguishable groups:

- **Polar:** a group favoring a water surroundings, containing arginine, aspartic acid, glutamic acid, lysine, asparagine, glutamine and proline, as well as the backbone particles including glycine.
- **Apolar:** a group favoring the center of the membrane, containing alanine, isoleucine, methionine, leucine, phenylalanine and valine.
- **Aromatic:** a group favoring the interface region between the hydrophobic core of the lipid membrane and the water, containing histidine, tryptophan and tyrosine.
- **Neutral:** a group with no significant preference, containing cysteine, threonine and serine.

From the shapes of the potential of mean force across the lipid membrane a qualitative estimate is made of the required interaction energy to recreate these shapes. Thus, the general behavior of the sidechain particles is more important than the inclusion of chemical detail. For the polar group the interaction with water is favorable, so all cross interaction energies with water are set to 1.97 kJ/mol. The interactions with the interface region of the lipid membrane (the lipid head particles) is not unfavorable, but not very favorable either and is, therefore, set to 0.50 kJ/mol, except for glycine, whose interaction energy is set to 1.00 kJ/mol. The interactions with the hydrophobic core (the lipid tail particles) are unfavorable and, accordingly, are set to be repulsive only.

On the other hand with the apolar group the diversity between the residues is larger, so some distinctions have to be made. For all residues in the group the interaction with water is unfavorable and is therefore set to be repulsive. The interactions with the lipid tail particles is set to be 1.00 kJ/mol for methionine and 1.97 kJ/mol elsewise. The interactions with the lipid head groups are neither strong nor weak for most residues in this group and is therefore set to 0.50 kJ/mol, except for phenylalanine. Phenylalanine has a much wider potential of mean force and, thus, seems to have a rather favorable interaction with lipid head particles, bringing its interaction strength to 1.00 kJ/mol.

The aromatic residues, histidine, tryptophan and tyrosine, only favor the interface region, so their interaction strengths with the lipid head particles is set to 1.97 kJ/mol. All other environment interactions for this group are set to be repulsive only. The last of the four groups, the neutral group, has no preference for any region, and, therefore, all interaction strengths are set to 0.50 kJ/mol.

The only interaction class not discussed so far is the protein-protein interaction class. These interactions are derived from the work by Liwo *et al.* [40]. However, since Liwo does not only use Lennard-Jones potentials, but also potential functions based both on distance and orientation, some adaptations to the parameters are necessary. These alterations have been made iteratively in the initial stage of development of the CG protein model. Finally, the interactions of backbone particles with sidechain particles are estimated from the assumption of the backbone particles being hydrophilic.

**Figure 4:**
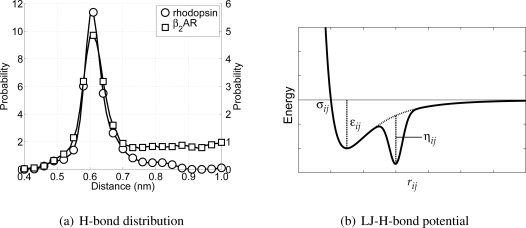
In (a) the distribution of potential H-bonding CG backbone particles along the backbone chain (*i* to *i* + 4) is depicted, whereas in (b) the newly developed Lennard-Jones H-bond potential is shown. The dashed line in (b) indicates the curve belonging to a regular Lennard-Jones potential. The important parameters are indicated in the figure.

As mentioned before, the new CG protein model excludes torsional interactions, but to account for the most important structural element in *α*-helices, H-bonds, some alterations to the non-bonded interactions are required. In regular *α*-helices the backbone particles which are above each other have the opportunity to form H-bonds. In terms of the chain sequence this means that the backbone particles on position *i* and *i* + 4 can form an H-bond, leading to an *α*-helix. Therefore the distance distributions for these backbone pairs have been determined from the previously used all-atom simulations. In Figure 4(a) these distributions are depicted. From these distributions it can be seen that there exists an average H-bond length of 0.61 nm (measured between the C*_α_*-atoms in the all-atom model). In order to maintain *α*-helices in the CG model, this distribution has to be reproduced. However, this is not possible using only the Lennard–Jones potential, because the distance for which the interaction energy is lowest is equal to 0.448 nm.

To overcome this problem an alternative type of the Lennard-Jones potential has been developed, which is only active between backbone particles. The alternative description consists of a regular Lennard-Jones potential, *V*_LJ_(*r_ij_*), combined with an inverted Gaussian curve, *V*_HB_(*r_ij_*), giving *V*_LJHB_(*r_ij_*) = *V*_LJ_(*r_ij_*) + *V*_HB_(*r_ij_*), with *V*_LJHB_(*r_ij_*) the Lennard–Jones H-bond (LJHB) potential. The Gaussian curve accounts for the H-bond part of the potential, since it can be used to introduce a second minimum in the potential at the distance at which the backbone particles should be in case an H-bond is formed. The H-bond contribution is defined as

(6) $$V_{\text{HB}}(r_{ij})=-\eta_{ij}{\text{e}}^{-\frac{1}{2}{\left(r_{ij}-\mu_{ij}\right)}^{2}/\kappa_{ij}^{2}}$$

where *μ_ij_* is the location of the H-bond minimum, *κ_ij_* determines the width of the H-bond well, and *η_ij_* represents the well depth of the H-bond minimum. In Figure 4(b) a schematic representation of the LJHB potential is shown. Both parameters *μ_ij_* and *κ_ij_* can be determined from the distribution shown previously in Figure 4(a), and in the case of backbone-backbone H-bond interactions these are 0.61 nm and 0.015 nm, respectively. The depth of *V*_HB_(*r_ij_*) is determined from computing the Coulomb contributions of the atoms the backbone particles represent involved in the H-bond maintaining the *α*-helix. Using the same all-atom MD simulations, the Coulomb energies for all of these possible H-bond forming pairs have been computed at the level of the CG particles. The parameter *η_ij_* is chosen in such a way that the well depth of the LJHB potential is in agreement with the computed values for the original Coulomb interactions. This gives for the well depth parameter of the backbone-backbone interaction an energy of 15.0 kJ/mol. It is important to realize that this LJHB potential is only active between CG backbone particles. Furthermore, the LJHB potential acts between any pair of backbone particles (except for those separated by one or two bonds). Thus, the LJHB potential has the ability to form *α*-helices, but only if the backbone particles are close to the hydrogen bonding distance *μ_ij_*. Otherwise, it simply acts as a normal Lennard-Jones potential.

All energies associated with all non-bonded interaction classes discussed above (protein-protein, environment-protein and environment-environment) are listed in Table S4 in the supplementary materials.

### 2.4. Comparison to Existing CG Protein Force Fields

In the previous section a complete description of the new CG protein model is given. However, this is not the first type of CG protein model that has been developed; over the past few years all kind of different force fields have been reported in literature.

The first CG protein models that have been developed mainly focused on the problem of protein folding [38–40,43,48]. The modeling of these folding processes is very complex and computationally time demanding, and, therefore, using CG models can be very beneficial. Recently, different CG models have been used to investigate protein folding again [30,31,49]. In most of these models the backbone of the protein is represented by all of its heavy atoms along the chain, which is a clear difference with the model introduced in this paper, which only has CG backbone particles at the C*_α_* positions. On the other hand, the sidechain particles are modeled as one CG particle as well.

A model that uses one CG particle for the backbone and one for the sidechain is the model developed by Voth *et al.* [32,50,51]. From this point of view it is very comparable to the present model. However, Voth *et al.* use the force matching algorithm [32,52,53] to determine free-form potentials from all-atom simulations for all interactions between the CG particles. The derived free-form potentials are then used as tabulated input for their model. Although this method allows for rapid determination of an underlying force field, it has to be derived again from all-atom simulations for every new molecular structure, for every new mixture and for every new state point.

The CG protein model which currently receives most attention is the model developed by Marrink *et al.* [33]. Recently Bond *et al.* have used this model to investigate peptide insertion into a membrane, as well as the behavior of membrane channel proteins [37,54–56]. Shih *et al.* have used Marrink’s model to take a closer look at discoidal lipoprotein particles [36]. A model similar to Marrink’s has recently been proposed in a study by Han *et al.* where they investigate poly-alanine based peptides [57].

In Marrink’s model the protein backbone is mapped into one CG particle as well, but the sidechains are not necessarily represented by one sidechain particle. For instance, the sidechain parts of arginine and lysine are modeled by two CG particles, whereas the ring based residues have either three or four CG particles. Moreover, besides glycine, also alanine is modeled as a backbone particle only. The difference between our and Marrink’s model is not only in the number of CG particles, but also in their size. Whereas the CG particles in the new model all have different sizes, depending on their occupied volumes, in Marrink’s model all particles have the same diameter (0.264 nm), except for the ring based particles (0.241 nm). Similarly, in Marrink’s model the masses for all CG particles are the same (72 amu), with the ring based particles (45 amu) as an exception again. Furthermore, instead of having different interaction types for each residue, in the model of Marrink all coarse grained amino acids are made of the similar building blocks as used in their lipid model [24], and, thus, the same coarse grained particle types are used to describe both lipids and amino acids. This is contrary to the present model, where different particle types are used for lipids and amino acids.

Although in our model 20 different particle types are necessary to describe the residues, Marrink’s model uses about 15 different building blocks to create the CG representation of all 20 residues. So the number of different particle types between both models is comparable leading to a similar complexity.

The major difference with respect to the modeling of the bonded interactions between Marrink’s model and our model is that Marrink included torsion interactions combined with backbone particle typing to fix the protein’s local secondary structure, while we discard torsion interactions explicitly. In a preliminary version of their model artificially bonds were added to constrain the conformation and orientation of *α*-helices [58]. In our model the secondary structure has to arise from the conformational freedom allowed by the double angle and the LJHB potentials.

Because Marrink’s model generally uses smaller and more particles to represent the CG sidechains, their force constants for bonds are smaller than the bond force constants in our model, which has larger and softer particles, but, consequently, needs to reduce the flexibility of the bonds. Furthermore, to avoid numerical instabilities arising from fast fluctuations, some backbone-sidechain bonds in Marrink’s model are constrained.

The different limitations between each of these CG protein models show that the applicability of a specific model might be limited to specific cases.

## 3. Performance Analysis of the Computational Model

To investigate the performance of the CG model two different series of MD simulations are carried out, the first being an analysis of the protein-environment non-bonded interactions, and the second a set of five different large protein complex simulations, each simulated for tens of nanoseconds.

**Figure 5:**
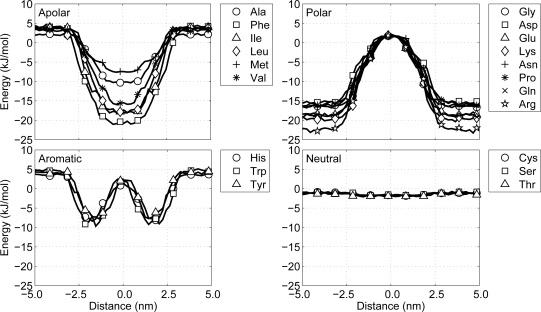
Non-bonded interaction energy between the CG protein particles and the environment (lipids and water) as a function of the distance to the membrane center. The protein particles are denoted by their common abbreviations and are put into their respective interaction groups: apolar, polar, aromatic and neutral, see the text.

Evaluating whether the non-bonded interactions between the protein and the environment correspond to the potentials of mean force in the model by Ulmschneider [47] is important. Therefore, MD simulations for each protein particle type (see Table S1) being pulled through a lipid membrane are performed. The lipids are modeled as H4T4T4-molecules (representing DPPC), which means that each lipid molecule has two tails of 4 T-particles each and a head group of 4 H-particles. These so-called steered MD simulations differ from normal MD simulations with respect to the fact that particles (in this case the protein particles) are pulled slowly but steadily through the simulation box. However, the particles themselves are not directly pulled, but instead they are connected to a phantom particle with a harmonic spring. The phantom particle is then pulled at a constant velocity and, through the spring, the true particle is pulled forward. The velocity at which this induced pulling occurs depends on the spring’s force constant, the pulling velocity for the phantom bead, and the interaction of the particle with the environment. Thus, the protein particles are pulled from the water phase through the membrane, to the other side of the membrane, back into the water again. It is important to pull slowly in order not to disturb the equilibrium of the system too much. Therefore, in all of these simulations the phantom particles are pulled at 0.002 nm/ps, which is two orders of magnitude below average thermal fluctuations. The force constant of the harmonic spring is set at 100 kJ/mol/nm^2^. The MD simulations are performed at constant pressure (1 atm) and constant temperature (324 K), using a Berendsen barostat and thermostat, respectively, and the size of the time step in the leapfrog integration scheme equals 12 fs. During the simulations the non-bonded interaction energy between the protein particle and the lipid-water environment is recorded as a function of the distance to the center of the membrane, see Figure 5. A closer look at these potential energy graphs shows that with the current model all particles behave similar with respect to the potentials of mean force presented by Ulmschneider. This indicates that protein-environment interactions in the CG protein model are as expected.

More recently, others have also performed similar experiments to obtain potential energy graphs with their CG protein model [33,59,60]. Comparing these profiles with ours shows different behavior for several residues, especially for the neutral residues in our model. However, whereas their models aim to include as much chemical detail as possible in the CG model, our model aims to model the general behavior, and, thus, using the implicit potentials based on the occurrence of residues with respect to the membrane is appropriate.

**Figure 6:**
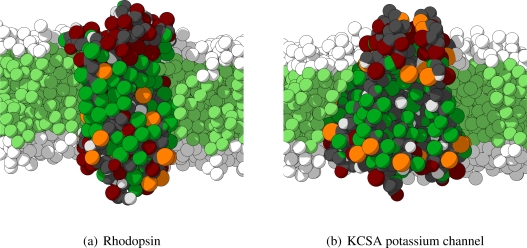
Initial configuration for the CG MD simulations for rhodopsin (on the left) and the KCSA potassium channel (on the right). Both proteins are embedded in an H4T4T4 lipid membrane, for both configurations only the part surrounding the proteins is shown and water and some lipids have been removed for clarity.

As mentioned previously, in order to analyze the behavior of the CG protein model at a larger level, five different large protein complexes are simulated for tens of nanoseconds. These five proteins are selected to cover a wide variety of size, shape and function to serve as test cases, and, therefore, are members of two different classes of proteins. Three of the selected proteins are transmembrane proteins (rhodopsin, *β*_2_AR, and the KCSA potassium channel) and two are water-soluble (cytochrome P450-CAM and fasculin 1). The crystal structures for each of these proteins are obtained from the protein data bank and converted into their respective CG representations. The water-soluble proteins are solvated in water only, whereas the transmembrane proteins are inserted in a periodic lipid membrane of appropriate thickness, depending on the length of the transmembrane region of the protein, and thereafter solvated in water. Hence, rhodopsin, the *β*_2_AR and the KCSA potassium channel are inserted in a membrane consisting of H4T4T4-molecules, having an approximate area of 19 × 19 nm^2^. Two important remarks have to be made. First, the four independent subunits of the KCSA potassium channel are not connected by any bonds, and are thus treated as independent molecules in the simulations. Secondly, some sections of the proteins are not included in the crystal structures and are, therefore, not included in the CG model. In particular for rhodopsin many of the non-helical intra- and extracellular regions are left out. In Figure 6 the starting conformations for rhodopsin and KCSA potassium channel are shown.

In all of the five MD simulations a timestep of 12 fs is being used, allowing for sufficient sampling of the fastest oscillations in the CG model (those in the bonded interactions), for a total simulation time of approximately 60 ns. Both the temperature and the pressure are kept constant (at 324 K and 1 atm respectively).

Whilst taking into account the different nature of each protein, all five simulations are analyzed in a similar fashion. For every protein the coordinate root mean square difference of the C*_α_*-atoms (CRMS*_α_*) with respect to the initial structure, and the change in radius of gyration (*R*_g_) are computed. Moreover, for each of the proteins the bond and angle distributions of the backbone particles are determined.

In Table 1 the CRMS*_α_* is shown for each of the simulations. This CRMS*_α_* is an average over the last 240 ps of the 60 ns simulation, and the crystal structure is used as a reference point. For KCSA two values are shown, one for the individual subunits and one for the entire complex, which takes the internal motions of the subunits into account. When the CRMS*_α_* is compared to the characteristic length scale of the model (being the backbone bond length of 0.384 nm), four out of five proteins appear not to change their conformations considerably. Only the entire KCSA-complex shows somewhat more conformational change. However, since the independent subunits of the KCSA potassium channel give a much lower CRMS*_α_* value than the total complex, it is apparent that the subunits have moved with respect to each other, leading to a higher CRMS*_α_* value. The results for the CRMS*_α_* suggest that the overall structure of these proteins is preserved reasonably well in the CG model.

Besides the CRMS*_α_* also the radius of gyration, *R*_g_, is shown in Table 1 for both the initial and the final conformation of the proteins. The radius of gyration is computed by taking the root mean square distance for all individual protein particles with respect to the geometric center of the protein [61]. From the results it can be seen that in general the radius of gyration decreases during the simulations. However, the amount of decrease is not the same for all proteins and, apparently, depends on the nature of the protein. The water-soluble protein fasculin 1 (a small randomly coiled protein) shows a small decrease (7%) in its radius of gyration. The only protein that has a relatively large decrease of its radius of gyration is cytochrome P450-CAM. However, this protein contains *α*-helices as well as *β*-sheets and random coils, which can explain the reduction in the radius of gyration. The other three transmembrane proteins (rhodopsin, *β*_2_AR and KCSA potassium channel) have a decrease in their radius of gyration of less than 5%. Probably these proteins are less compressible than the other two.

For further evaluation of the performance of the CG protein model, the results of the CG simulations are compared to those from all-atom MD simulations for two proteins: rhodopsin and *β*_2_AR. The simulations of these two are already available (and have been used before [8,41]). For each of the two all-atom simulations a trajectory of 1 ns is used for analysis, with snapshots taken every several picoseconds.

**Figure 7:**
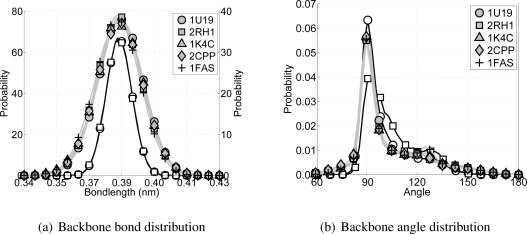
In (a) the backbone bond distributions for both all-atom simulations (black lines) and the five CG simulations (gray lines) are shown. Using a similar color scheme the backbone angle distributions are shown in (b). The all-atom simulations are on the left axis, and, if applicable, the CG simulations on the right axis. The protein data bank codes for the proteins are used in the legend, see Table 1.

¿From each of the CG simulations, as well as from each of the all-atom simulations, the bond distributions for the bonds between backbone particles and the angle distributions within a triplet of backbone particles are computed, see Figure 7. With respect to the bond distributions it is clear that the distributions for the CG simulations are slightly wider than for the all-atom simulations, but still acceptable. For the angle distributions along the backbone it can be seen that the all-atom simulations have a high peak around 91° and a shoulder around 123°, which coincides with the expected locations for the *α*-helices on one and the loops and random coils on the other side. In the CG model the backbone angle interaction is governed by the double angle potential. The parameters for this potential are chosen to mimic the *α*-helices of transmembrane proteins best. ¿From Figure 7(b) it can be seen that the angle distribution for all CG simulations are similar to the all-atom angle distributions for the transmembrane proteins rhodopsin and the *β*_2_AR.

When developing the CG model special attention has been paid in recovering the secondary structure in the model, without imposing too much knowledge about the secondary structure upon the model. Thus, the performance of the model can partially be measured by the correct representation of the protein’s secondary structure. Since the model is aimed at reproducing *α*-helices in transmembrane proteins, several non-bonded backbone particle distances (or C*_α_* distances in the all-atom simulations) along the backbone chain are investigated for the rhodopsin and *β*_2_AR simulations. Because a regular *α*-helix is formed when there is an H-bond between particle *i* and its neighbor on position *i* + 4, this distance is tracked throughout the simulations. Also the distances are monitored between particles which can form H-bonds leading to tighter and wider helices (*i* to *i* + 3 and *i* to *i* + 5).

**Figure 8:**
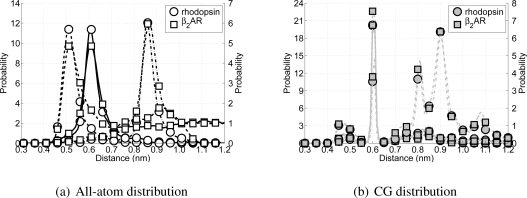
Distributions of potential H-bond forming pairs in the all-atom simulations (on the left) and in the CG simulations (on the right). For the possible H-bond forming pairs of rhodopsin and the *β*_2_AR backbone particles along the chain are considered, where the dashed lines indicate *i* to *i* + 3 pairs, solid lines *i* to *i* + 4 pairs, and dash-dotted lines *i* to *i* + 5 pairs. In the left figure the distribution for *β*_2_AR is put on the right y-axis, whereas in the right figure the distributions for the *i* to *i* + 5 pairs are on the right y-axis.

All possible H-bond distributions are shown in Figure 8. ¿From the all-atom distributions (Figure 8(a)) it can be seen that rhodopsin and the *β*_2_AR have similar H-bond distributions for all possible H-bond forming pairs (*i* + 3 to *i* + 5). The only difference arises from the fact that in the all-atom model of the *β*_2_AR more intra- and extracellular loops are present, which arises in the figure as longer tails for each of the three distributions. Turning the attention to the CG model for these two proteins and their respective potential H-bond forming pairs (see Figure 8(b)) a clear peak can be seen around 0.61 nm, which coincides with the distance *μ_ij_* in the Lennard-Jones H-bond potential. However, while the peak for the *i* to *i* + 5 pairs is still present, albeit with a much lower probability, the peak for the *i* to *i* + 3 pairs has moved to 0.61 nm as well. Although the Lennard-Jones hydrogen bond potential is not active between backbone particles that are bonded or connected directly with an angle, it is active between the *i* to *i* + 3 pairs, favoring these pairs to be at the ideal distance for the H-bond part of the Lennard–Jones H-bond potential. On the other hand visual inspection of both trajectories showed that the structure of these CG proteins is still helical, although the helices are slightly tighter than true *α*-helices. However, this does not significantly affect the lengths of the helices, nor does it cause the sidechain particles to point inward toward the backbone helix. Therefore, the current representation of the CG *α*-helix is considered acceptable.

Concluding the investigation of the performance of the CG model, it has been shown that proteins with *α*-helices can be modeled quite accurately. Furthermore, the protein-environment interaction energies are reproduced satisfactorily, as are the bond and angle distributions.

## 4. Case Study: WALP-peptides

With respect to the structure and function of biological membranes the interaction between proteins and lipids is crucial. An important factor of this interaction is matching the hydrophobic thickness of the membrane with the hydrophobic length of the transmembrane protein segment. Processes that can be influenced by this hydrophobic matching include protein activity, membrane domain formation and protein sorting [62,63]. Therefore, in order to understand the effects of hydrophobic matching it is important to have detailed information at the molecular level.

Because large protein complexes embedded in lipid membranes are difficult to study both experimentally and theoretically, small model systems to understand the effect of hydrophobic matching have been introduced which mimic the transmembrane segments of membrane proteins. The best known example of these model systems is a family of synthetic *α*-helical transmembrane model peptides, having a repeated alanine-leucine-sequence flanked on both sides by tryptophan residues, called WALP-peptides [63–67]. The flanking tryptophan residues have been chosen because these residues are frequently found in membrane proteins near the membrane interfacial region and because they orient with the lipid headgroups [68]. At room temperature the helical axis of these WALP-peptides is nearly perpendicular to the plane of the membrane and, the peptide being membrane embedded, strongly protected from the solvent [69]. Furthermore, because these hydrophobic peptides form well-defined *α*-helices, as has been proven by several experiments [63,66,67,70–72], they are suitable to investigate the packing of *α*-helices in transmembrane proteins, such as G-protein coupled receptors [73].

The previously mentioned experimental studies have been complemented by all-atom MD simulations investigating the properties of these peptides and their folding into the membrane [74–77]. More recently, MD simulation studies of the insertion of WALP-peptides into the lipid membrane have been performed using an adapted version of Marrink’s CG model [37].

To show the applicability of the currently presented CG model for transmembrane proteins, MD simulations of WALP-peptides of different length embedded in lipid membranes of different thickness are performed. Fourteen peptides of systematically increasing length are used, denoted as WALPx, where x is the number of residues in the peptide, ranging from 16 to 41. For example, the amino acid sequence of WALP16 is GWWLALALALALAWWA. For longer peptides the LA-sequence is extended.

**Figure 9:**
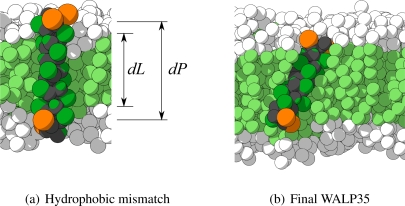
Configurations for different WALPs. In (a) the initial configuration for WALP27 embedded in an H4T3T3 lipid membrane is shown, with the thickness of the hydrophobic part of the membrane (*dL*) and the hydrophobic part of the peptide (*dP*) indicated. In (b) the final configuration for WALP35 in an H4T4T4 lipid membrane are depicted. In each figure water and some lipids are removed for clarity.

To create the starting conformations for each of the peptides, first the all-atom *α*-helical representation has been built. Subsequently, these all-atom representations have been converted into their CG counterparts. The peptides are then combined with two different lipid membranes, either dilauroylphosphatidylcholine (DLPC) or DPPC, represented in the CG model by H4T3T3- and H4T4T4-molecules respectively. The two different membranes are chosen to expose all WALP-peptides to different membrane thicknesses. Finally, the entire system is solvated in water. The area for the lipid membrane is approximately 10 × 10 nm^2^. An example of the CG representation of WALP27 (with water omitted for clarity) is shown in Figure 9(a). In this figure also the definition of the hydrophobic mismatch is depicted, which is the difference between the length of the hydrophobic part of the peptide (*dP*) and the thickness of the hydrophobic part of the membrane (*dL*). A negative hydrophobic mismatch means that the hydrophobic part of the peptide is shorter than the hydrophobic part of the membrane, and a positive hydrophobic mismatch the opposite.

With fourteen different WALP-peptides in two different lipid membranes, at least 28 CG MD simulations are to be performed. However, to improve statistical accuracy, all simulations are performed three times. In each of these three repeated simulations the initial conformations are the same, but the initial velocities are changed. Furthermore, all simulations are performed for 24 ns, with a time step size of 12 fs. Both the temperature and pressure are kept constant around atmospheric conditions, again using the Berendsen coupling schemes. All simulations are performed 10 K above the melting temperature of the lipids in the membrane, which equals 293 K for H4T3T3 and 324 K for H4T4T4. Unless mentioned otherwise all presented results are the averages of the three repeated simulations.

To verify whether the structure of the WALP-peptides remains intact after the CG MD simulations, the CRMS*_α_* is computed for each peptide with respect to its initial configuration. In Table 2 these values are shown for each peptide in both type of membranes. For most simulations the CRMS*_α_* is very low, with values below 0.2 nm for 22 out of 28 peptides. Moreover, all peptides have a CRMS*_α_* which is below the previously used threshold of 0.384 nm (the characteristic length scale of the model). Visual inspection of the few peptides that have a somewhat larger CRMS*_α_* revealed that each of the peptides still maintained its *α*-helical character. In Figure 9(b) the final configuration of the peptide with the highest CRMS*_α_* (WALP35 in H4T4T4) is depicted, showing indeed the *α*-helix being maintained. The most probable cause for its higher CRMS*_α_* is a small bend that appeared in the *α*-helix.

The stress introduced in a lipid membrane by a hydrophobic mismatch due to an inserted peptide can be relieved by at least two mechanisms [78,79]. The first mechanism involves the membrane adapting its local thickness to match the length of the hydrophobic part of the peptide, while the second mechanism is the tilting of the peptides to decrease any positive hydrophobic mismatch. Obviously a negative hydrophobic mismatch cannot be removed by tilting, because tilting would decrease the hydrophobic mismatch even further. Using the CG MD simulations for the WALP-peptides it is possible to investigate the occurrence of both mechanisms.

In order to compute the local membrane thickness the entire membrane is first divided into a 17 × 17 grid of equally sized sections. For each of these 289 sections the membrane thickness is computed separately by measuring the distance of the tail particles with respect to the central membrane plane of that section. For each distance computation of each lipid only the tail particles connected to the head particles are used. Multiplying the obtained distance for each tail particle by two gives the local membrane thickness of that lipid. Averaging for all lipids in a section gives the membrane thickness of that specific part. For both lipid membranes (H4T3T3 and H4T4T4) the same procedure is used. Because the membrane thickness is now known throughout the entire 17 × 17 grid, it is possible to compute the membrane adaptation due to the insertion of the peptide. This adaptation is defined as the difference between the membrane thickness near the peptide and far away from the peptide. It is observed that for smaller peptides the membrane adaptation is negative, it increases as the peptide length increases, and eventually becomes positive.

Subsequently, the hydrophobic mismatch is computed based on the thickness of the membrane far away from the peptide (*dL*) and the hydrophobic length of the peptide (*dP*). Because in each WALP-peptide the outer amino acids are non-lipophilic, the hydrophobic length of the peptide is assumed to be the distance between the average positions of the four N-terminal and the four C-terminal backbone particles, which should more or less coincide with the beginning of the hydrophobic part of the peptide.

**Figure 10:**
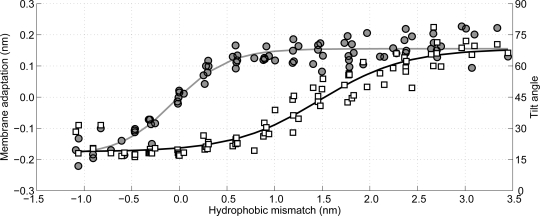
The effect of hydrophobic mismatch on peptide tilt and membrane adaptation. The membrane adaptation is shown by the circles and indicated on the left vertical axis, while the peptide tilt is on the right vertical axis and shown with squares. The lines are sigmoid curves fitted to the data and are only to guide the eye; both lines have a regression coefficient of 0.93.

In Figure 10 the membrane adaptation due to the peptide insertion is shown as a function of the hydrophobic mismatch (circles, left axis). As can be observed there is no membrane adaptation when the hydrophobic mismatch is zero. Moreover, when the hydrophobic mismatch decreases the membrane becomes thinner around the peptide, whereas when the hydrophobic mismatch increases the membrane thickens, until a plateau is reached (approximately 0.15 nm of membrane adaptation). Hence, the first proposed mechanism of relieving the stress introduced by the hydrophobic mismatch is confirmed.

The tilting of the WALP-peptides can be computed by determining the angle between the helical axis and the normal to the membrane, where an angle of 0° coincides with a perfect transmembrane alignment. Because all membranes are located in the *xy*-plane, the membrane normal is chosen to be the *z*-axis of the system, which is accurate enough as long as the local oscillations of the membrane remain small. The helical axis is computed using the same terminal residues as used for the computation of the hydrophobic length of the peptide. This definition is expected to be accurate for *α*-helical peptides, such as WALP-peptides. In Figure 10 the tilt angle of the WALP-peptides is also shown, again as a function of the hydrophobic mismatch (squares, right axis). For the peptides with a negative hydrophobic mismatch the tilting varies between 15° and 30°, which can be assumed to be due to diffusion-driven stochastic movements. When the hydrophobic mismatch increases the peptide tilt also increases gradually, up to a tilt of approximately 70°. The tilt angles computed for the WALP-peptides of intermediate length (WALP19, 21, 23, 25) correspond well with previously reported tilt angles from other simulations [37,79–81]. However, experimental tilt angles are much lower than those computed from MD simulations, due to an averaging effect caused by dynamic variations of the tilt and rotation of the peptide [81,82]. It can be shown that when applying the same averaging procedures, which occur within the experiments, to the MD simulations, the same tilt angles are recovered [82]. Thus, it is shown in the simulations that peptide tilting is a mechanism of relieving stress from a hydrophobic mismatched membrane.

One of the most interesting observations that can be made from Figure 10 is that the membrane adaptation occurs at a lower hydrophobic mismatch than the peptide tilting. Moreover, it seems that first the membrane adaptation is used to decrease tension from the hydrophobic mismatch, followed by tilting of the peptides when the membrane can no longer adapt its thickness easily. Although both mechanisms for stress relaxation have been proven previously by others [79], from the current work it can be seen that both mechanisms occur sequentially and not in parallel, with the membrane adaptation coming first.

Although the membrane adaptation has been shown to be the first step in dealing with the introduced stress in the membrane due to the hydrophobic mismatch, this could be an artifact caused by the force field when the lipids are not modeled rigid enough. Therefore, four different WALP-peptides (WALP19, 23, 27 and 31) are put in an H4T3T3-membrane where the force constant belonging to the angles in the lipid tails is doubled (from 5.41 to 10.82 kJ/mol/rad^2^). By choosing these peptides and this membrane a range for the hydrophobic mismatch from 0 to 1.8 nm is used for further investigation. The consequence of the increased force constant is that the lipids become much more rigid and, hence, deformations and fluctuations in the membrane are less likely to occur, and, also, the membrane thickens.

**Figure 11:**
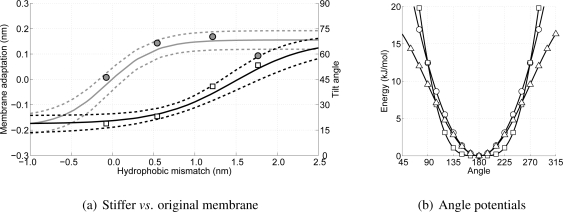
In (a) the data shown in Figure 10 is repeated using only the sigmoid curves (solid lines). The dotted lines are used to indicate the bandwidth (plus or minus the standard deviation) for both the membrane adaptation (left axis) and the peptide tilt (right axis) as a function of the hydrophobic mismatch. The circles and squares are the values computed from the stiffer membrane. In (b) three lipid tail angle potentials from different CG models are shown (a square denotes Marrink’s model, a triangle Klein’s, and a circle the current model).

In Figure 11(a) the results for the membrane adaptation and peptide tilt angle as a function of the hydrophobic mismatch for these four WALP-peptides are shown. In this figure also the progression of the membrane adaptation and peptide tilt angle for the original simulations is indicated using the (dashed) lines. From this figure it can be seen that the peptide tilt (indicated by the squares) is not significantly different, and also the membrane adaptation is in good agreement with the previous simulations. Only in the case of the higher hydrophobic mismatch the membrane adaptation shows some deviation. However, although the found value lies outside the bandwidth region (plus or minus the standard deviation), from Figure 10 it can be seen that at similar hydrophobic mismatches membrane adaptations ranging from 0.1 to 0.2 nm are found, and, thus, the value for the stiffer membrane is believed to be at least similar to the case with the normal membrane. Consequently, the stiffer membrane has no apparent effect on the order of occurrence of the two mechanisms to reduce stress in the membrane due to peptide insertion, and, thus, these mechanisms are thought to be a genuine property of peptide-lipid interactions.

Comparing the angle potential for the lipid tails (with the original force parameter of 5.41 kJ/mol/rad^2^) with other force fields, such as Marrink’s or Klein’s [20,24], it can be seen that all of these are in good agreement, see Figure 11(b). Therefore, changing the force constant by as much as 100% does not seem plausible, and confidence in the observed two-step mechanism in dealing with the hydrophobic mismatch is strengthened once more.

In this case study the newly developed CG model for transmembrane proteins showed that it is applicable to investigate model peptide systems, such as WALP-peptides. Moreover, the model showed that when dealing with a positive hydrophobic mismatch, the peptide-membrane system first thickens the membrane around the peptide before the peptide is tilted in order to reduce the hydrophobic mismatch.

## 5. Conclusions

In this paper the first version of a CG protein model aimed at transmembrane *α*-helical proteins is described. The main philosophy of coarse graining in this model is based on existing models, for instance the size and mass of the CG particles, but also new interaction potentials are introduced, for example the double angle or the Lennard-Jones H-bond potential. Compared to other CG protein models the main difference with the presented model lies in the number of CG particles used for each side chain particle, with the omission of torsional interactions, and the introduction of new potentials. This last step is a different approach to CG modeling, but in our view a route worthwhile following.

Before showing the applicability of the model, first a performance analysis is conducted, in order to show that the model behaves as expected. To that end it is shown that for each of the CG sidechain particles its preferred location in a lipid membrane is consistent with previously published results, showing that the non-bonded interactions that govern this preference are correctly modeled.

Furthermore, five very different protein complexes have been investigated using MD simulations for several tens of nanoseconds. In general the CRMS*_α_* for these proteins are within an acceptable range, indicating the CG model performs well. A closer look at the radius of gyration of the protein complexes confirms this.

For each of the five protein complexes the bond and angle distributions are compared to all-atom MD simulations. Besides the intrinsic property of coarse graining to give rise to softer potentials compared to all-atom potentials, which leads to broader distributions, both all-atom and CG distributions agree very well. However, the possible H-bond forming pairs in both the all-atom and CG representations of two out of five protein complexes shows that the CG model strongly prefers an H-bond distance of 0.61 nm, even for preferred H-bond distances in the all-atom model of around 0.5 nm. The investigation of the performance of the CG model shows that the new model works reasonably well for proteins that are rich in *α*-helices, especially when keeping in mind that the aim has been the development of a CG model for transmembrane *α*-helical proteins.

The applicability of the model is shown by a case study for WALP-peptides. The WALP-peptides are used both in experiments and theory as a model system for the interaction between transmembrane peptides and lipid membranes. Using the CG protein model with MD simulations on WALP-peptides of different length and embedded in lipid membranes of different thickness, it is shown that the apparent hydrophobic mismatch between peptide and membrane can be resolved by two mechanisms. In the first mechanism the membrane adapts its thickness to accommodate the peptide in its transmembrane orientation. However, when it is no longer feasible for the membrane to adapt its thickness, the second mechanism forces the peptide to tilt with respect to the membrane normal. It is observed that both mechanisms occur sequentially and not in parallel.

In the future the currently presented first version of the CG protein model needs to be further improved, for instance with respect to protein aggregation, because a possible imbalance in the cross-type non-bonded interaction parameters could cause unwanted protein aggregation behavior. Furthermore, the H-bonding aspects of the current model could probably be improved by adding directionality to the Lennard–Jones H-bond potential, for instance by looking at the angle between H-bond forming pairs.

## Figures and Tables

**Table 1:** Analysis results for five different protein CG MD simulations. Both the CRMS*_α_* and *R*_g_ (for the initial and final conformations) are given. All numbers are an average over 240 ps of simulation time. For the KCSA potassium channel the results are given for the total complex as well as for an average of the separate subunits.

Protein	PDB-code	CRMS*_α_* (nm)	*R_g_*^init^ (nm)	*R_g_*^fin^ (nm)
rhodopsin	1U19	0.323 ± 0.004	2.23 ± 0.005	2.19 ± 0.005
*β*_2_AR	2RH1	0.382 ± 0.004	2.02 ± 0.005	1.97 ± 0.005
KCSA (total)	1K4C	0.511 ± 0.002	2.12 ± 0.005	2.03 ± 0.005
KCSA (individual)	1K4C	0.393 ± 0.003	-	-
cytochrome P450-CAM	2CPP	0.323 ± 0.002	2.13 ± 0.005	−1.97 ± 0.005
fasculin 1	1FAS	0.196 ± 0.003	1.12 ± 0.005	1.02 ± 0.005

**Table 2:** The CRMS*_α_* (in nm) after 24 ns of coarse grained molecular dynamics simulations for WALP-peptides of different length embedded in two different lipid membranes. The presented values are an average of the final 240 ps of the simulations, and for each of the simulations the standard deviation for the CRMS*_α_* equals 0.005 nm.

	WALPx													
Lipid	16	17	19	21	23	25	27	29	31	33	35	37	39	41
H4T3T3	0.092	0.054	0.045	0.038	0.061	0.143	0.109	0.045	0.080	0.131	0.191	0.179	0.151	0.126
H4T4T4	0.052	0.188	0.184	0.208	0.048	0.084	0.209	0.241	0.162	0.146	0.323	0.174	0.266	0.261
